# Fatal Traps in Western Barn Owls' (*Tyto alba*) Nesting Sites: The Consequence of Improper Building Modification

**DOI:** 10.1002/ece3.71409

**Published:** 2025-05-08

**Authors:** Zoltán Schneider, Balázs Móczár, Ákos Klein, Miklós Laczi, Róbert Mátics

**Affiliations:** ^1^ Doctoral School of Biology and Sport Biology, Faculty of Sciences University of Pécs Pécs Hungary; ^2^ The Barn Owl Foundation Orosztony Hungary; ^3^ Veszprém County Group of BirdfLife Hungary Balatonkenese Hungary; ^4^ HUN‐REN–ELTE–MTM Integrative Ecology Research Group ELTE Eötvös Loránd University Budapest Hungary; ^5^ Behavioural Ecology Group, Department of Systematic Zoology and Ecology ELTE Eötvös Loránd University Budapest Hungary; ^6^ Department of Behavioural Sciences, Medical School University of Pécs Pécs Hungary; ^7^ Hungarian Nature Research Society Ajka Hungary

**Keywords:** avian mortality, building renovation, church tower, human impact, nestbox, wildlife management

## Abstract

Human activities can highly impact species that rely on man‐made structures for shelter or nesting, and building modifications for pest control or maintenance can have hidden ecological consequences. The breeding of the western barn owl ( *Tyto alba* ; hereafter barn owl) is especially associated with agricultural and church buildings. It is well known that the renovation of churches and their architectural protection against unwanted animals have significantly reduced the availability of barn owl nesting sites in recent decades. However, other potential direct consequences of church modifications have not been investigated sufficiently. We demonstrated how improperly modified church towers can act as traps, allowing the owls to enter the building but preventing them from leaving, which can lead to mass mortality, as we documented in two cases. This study highlights the need for deeper and more multifaceted investigations of building protection in light of such consequences that have been generally hidden until now in order to protect the barn owl more effectively.

## Graphical abstract

We demonstrated how improperly modified church towers can act as traps, allowing the barn owls to enter the building, but preventing them from leaving, which can lead to mass mortality. This study highlights the need for deeper and more multifaceted investigations of building closure and maintenance in the light of such consequences that have been generally hidden until now, in order to protect the barn owl more effectively.

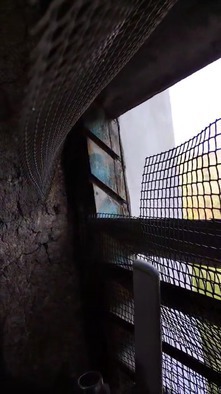

## 1 Introduction

The interaction between human infrastructure and wildlife is recognized as a crucial aspect of biodiversity conservation (e.g., Soulsbury and White 2015; Torres et al. 2016). Species associated with or dependent on man‐made structures with regard to reproduction or shelter face a range of challenges as human activities continuously reshape these structures. Architectural modifications to the structures, intended for pest control or maintenance, can have unforeseen ecological consequences that may threaten the species in an additional way; therefore, it is essential to exactly identify these risk factors.

Church buildings (hereafter churches) play a significant role in maintaining biodiversity (Skórka et al. 2018). Several European bat species use church attics and tower spaces as roosting sites (Zeale et al. 2016; Rydell et al. 2017), and churches also play a crucial role in maintaining local bird fauna (Skórka et al. 2018).

However, the use of buildings by wildlife sometimes leads to human‐wildlife conflicts (Stone et al. 2015; Zeale et al. 2016). In the case of churches, bats and feral pigeons (*Columba livia f. domestica*) are the primary sources of the conflict. Since feral pigeons often settle in large numbers in such buildings, these structures are frequently sealed off with bird‐proof netting to keep the pigeons out (Poprach 2010). However, this specialized pest control strategy generally leads to the exclusion of all other bird species as well.

The barn owl ( *Tyto alba* ) is one of the most characteristic birds associated with churches. A considerable proportion of the population in Central Europe still nests in this kind of building (Bank et al. 2019; Żmihorski et al. 2020; Klein et al. 2022). Architectural modifications to structures that barn owls use as nesting sites are widespread across Europe due to the significant rate of demolition and conversion of the most utilized buildings, like barns (Ramsden 1998), as well as the pest control and renovations of churches (Golawski et al. 2003; Poprach 2010; Klein et al. 2023). Golawski et al. (2003) found that the availability of churches for barn owls declined from 79% to 52% between the 1989 and 1992 period and the year 2000. In many European countries, the decline of barn owl populations is well documented (Martinez and Zuberogoitia 2004; Poprach 2010; Żmihorski et al. 2020), and the reduction in the number of suitable nesting sites due to the above‐mentioned factors may play a role in this decline.

With regards to barn owls, the most well‐known and leading non‐natural cause of mortality is collisions with vehicles across Europe (Newton et al. 1997; Mátics 1999, 2004; Fajardo 2001; Šálek et al. 2019). However, several other factors can also directly lead to individual fatalities, such as poisoning, collisions with electrical wires, drowning in water containers, or entrapment in vertical hollow objects or buildings (Fajardo 2001; Poprach 2010; Šálek et al. 2019). Among the anthropogenic causes of death in barn owls, vehicle collisions have been the most intensively studied, while there is considerably less knowledge available regarding the nature of other human‐induced mortality factors. Šálek et al. (2019), in a study conducted in the Czech Republic, found that entrapment in vertically hollow objects and drowning in liquid reservoirs are the second most common direct causes of anthropogenic mortality after roadkill. Fajardo (2001) also found that accidental entrapment in buildings occurs as a mortality factor, though only to a negligible extent.

In the present study, we report two cases that lead us to propose that man‐made structures functioning as nesting sites could potentially act as fatal traps when the buildings are modified improperly, posing an overlooked threat to barn owls. In the present study, the term “trap” refers to literal traps, distinct from ecological traps, where individuals select sink habitats (Battin 2004).

## 2 Observations

Since the 1980s, barn owl conservation efforts have been continuously carried out in several counties in Hungary (Kalotás 1987; Bank et al. 2019; Klein et al. 2023). These activities involve identifying nesting sites, installing nestboxes (mainly in church towers), checking nestboxes to collect breeding data, and raising awareness among building maintainers.

In 2021 and 2024 during the conservation work in Veszprém County, we found fresh barn owl carcasses in buildings protected by bird netting. Hence, these buildings were previously assumed to be closed for owls, as barn owls cannot gain access to the attic or church tower because all entry points, such as windows, are secured if the metal/plastic bird netting is properly installed and is in proper condition too.

On 22 February 2021, a site revisit was conducted at a church (47°06′53.6″N 18°08′05.0″E) where a nestbox for barn owls had been installed in the past, but no data had been recorded from the location for 20 years. In the nestbox, we found seven eggs on June 13, five nestlings and two eggs on 9 July, and six nestlings on 7 August when we ringed them. However, on 17 October, five of the six previously ringed chicks were found dead in a fully fledged developmental stage, along with one unringed adult (probably one of the parents) and another that had been ringed as a fledgling on 8 August at a site 27 km away. All seven owls in the building were dead and all carcasses were discovered in the tower and attic space. At this point, it is important to note that the nestboxes (placed in the window) are enclosed on the interior side of the building; therefore, owls have no direct access to the building interior from the nestbox. The building had been closed off years earlier by the maintainers, making it impossible for birds to access it. However, a thorough inspection revealed that a section of the mesh stapled on the inner side of the windows had partially detached. We presumed that the birds entered through this opening but were unable to exit, effectively turning the building into a fatal trap for them.

On 8 August 2024, in another church (47°23′26.3″N 17°40′14.7″E), we discovered the carcasses of nine barn owls in the attic. All nine birds fledged that year or were adults, but their exact age could not be determined from the carcasses. One of the birds wore a ring, which allowed us to identify that it had been ringed as a nestling in the summer of 2023, 7.5 km away. This case was very similar to the above described one, with the key difference being that no nestbox had been installed here. According to the building maintainer's report, the building had been closed off years ago. However, we still found a section of the wire mesh that had torn from the window, which is presumed to be how the birds gained access. Since no signs of nesting were found in the attic or tower, and the number of pellets was negligible, it is almost certain that the birds did not nest in the church and did not use the space for resting.

## 3 Discussion

When protecting buildings from unwanted animals, property maintainers typically use thin chicken wire or plastic mesh for the closures. Unfortunately, these kinds of pest‐control installations are not selective with regards to target species, leading to the exclusion of species that otherwise need protection and support. In addition, these installations are not always perfect and may have lethal consequences, as our case study shows. During survey work, we frequently observed that these materials were improperly secured, leading to their detachment. In the case of tall church towers, these closures are exposed to harsh weather conditions, which further compromise their effectiveness. Furthermore, plastic meshes, which are frequently used materials, can degrade more easily because of the pronounced photo‐oxidation of plastics by solar UV‐B radiation (e.g., Andrady et al. 1998).

We suggest that the cases we report here are the result of a highly overlooked aspect of pest‐control. The observed conditions lead us to conclude that the birds entered through a torn bird‐proof netting placed on the window but they were unable to exit. The partially torn net could function as a trap, allowing the birds to enter the building but preventing them from leaving (Video 1). Similarly to classic trap doors, birds can enter from the outside, as they can push it open. However, it is much less effective for them to exit, as in many cases, it is not possible or requires significant force to do so. Additionally, as shown in the video, wind can open and close the trap as well. Barn owls actively seek nesting sites and may enter these locations that initially appear suitable but from which they cannot escape. It is particularly dangerous because, in the first case, it was proven that most of the individuals originated from the same brood. Since barn owl parental care during the post‐fledging dependence period can last for several weeks (Taylor 2004; Béziers and Roulin 2016), and fledglings use vocalizations to call for food, it is plausible that if one young bird enters the building, its calls attract others, hence potentially increasing the number of victims caught in the trap. Alternatively, it would also be possible that the birds were not trapped, but rather killed by poison or a predator, but we can exclude these scenarios. First, according to the information provided by the building maintainers, no pest control had been conducted using toxic substances that could have potentially poisoned the owls. At the same time, it is possible that rodenticides or other toxins from food contributed to the deaths of the birds. The home range of a male barn owl can reach up to 19.8 km^2^ (Séchaud et al. 2022), allowing it to gather poisoned prey from a very large area. However, given the circumstances, we consider it unlikely that multiple fledged birds would die approximately simultaneously in the same building without any evidence of continuous use of the location. Second, there were no external injuries or other typical signs of predation on the birds.

**VIDEO 1:** The video demonstrates how an improperly secured net can act as a trap. Even the movement of the wind can open or close the net. It can be easily pushed inward by an owl, but getting out is very difficult. Video content can be viewed at https://onlinelibrary.wiley.com/doi/10.1002/ece3.71409

Poprach (2010) presents several cases in which barn owls become trapped in buildings. In most of these instances, the building was intentionally or accidentally closed while the owls were still inside. If there are signs of nesting or a large accumulation of pellets, it can be easily concluded that the owls have been using the building continuously and that its maintainers may have inadvertently locked them in. However, if there are no signs of nesting or pellets, it is more likely that the owls entered the building accidentally on a single occasion. In the cases we have presented, the latter scenario was most likely the case.

Barn owls are secondary cavity‐nesters. However, due to the declining number of suitable hollow trees, they now primarily utilize the cavities that human structures provide (e.g., church steeples, barns, attics) in many areas, particularly in Europe (Taylor 2004). In Central Europe, a considerable portion of the population still nests in churches (Bank et al. 2019; Żmihorski et al. 2020; Klein et al. 2022), either in nestboxes installed in the tower or in the spire space. The decline in nest site availability caused by the renovation and closing of churches likely makes the remaining accessible nesting sites even more important. In this context, it can be assumed that barn owls are forced to rely on the few opportunities available to them. As a result, improperly closed buildings can become fatal traps.

Based on this, if possible, we recommend reopening the churches in such a way that the attic and the tower are freely accessible to the barn owls. This is likely the best solution for the barn owls (Klein et al. 2007). If this is not feasible, the nest boxes placed in the tower could also provide suitable nesting sites for the species (Bank et al. 2019). If no suitable nesting sites for the species can be provided within the building, they should be properly closed to prevent access and accidents. These closures should be carried out only in collaboration with and under the supervision of the relevant conservation authorities. Furthermore, it should be considered to install one‐way security doors that allow the bird to exit the building but prevent entry. This way, a bird that has accidentally entered through an insecure net can still exit the building. In conclusion, our study highlights a neglected risk factor affecting a species in rapidly changing human environments and advocates for improved monitoring and management to support barn owls more effectively.

## Author Contributions

**Zoltán Schneider:** conceptualization (lead), investigation (equal), project administration (equal), writing – original draft (lead), writing – review and editing (equal). **Balázs Móczár:** conceptualization (equal), investigation (lead), resources (equal), visualization (equal), writing – original draft (equal). **Ákos Klein:** project administration (equal), resources (equal), supervision (equal), writing – original draft (equal), writing – review and editing (equal). **Miklós Laczi:** conceptualization (equal), methodology (equal), supervision (lead), writing – original draft (equal), writing – review and editing (lead). **Róbert Mátics:** conceptualization (equal), project administration (equal), resources (equal), supervision (equal), writing – original draft (equal), writing – review and editing (equal).

## Conflicts of Interest

The authors declare no conflicts of interest.

## Data Availability

There are no additional data, only observations that are described in the article.

## References

[ece371409-bib-0001] Andrady, A. L. , S. H. Hamid , X. Hu , and A. Torikai . 1998. “Effects of Increased Solar Ultraviolet Radiation on Materials.” Journal of Photochemistry and Photobiology B: Biology 46, no. 1–3: 96–103. 9894353 10.1016/s1011-1344(98)00188-2

[ece371409-bib-0002] Bank, L. , L. Haraszthy , A. Horváth , and G. F. Horváth . 2019. “Nesting Success and Productivity of the Common Barn‐Owl *Tyto Alba*: Results From a Nest Box Installation and Long‐Term Breeding Monitoring Program in Southern Hungary.” Ornis Hungarica 27, no. 1: 1–31. 10.2478/orhu-2019-0001.

[ece371409-bib-0003] Battin, J. 2004. “When Good Animals Love Bad Habitats: Ecological Traps and the Conservation of Animal Populations.” Conservation Biology 18, no. 6: 1482–1491. 10.1111/j.1523-1739.2004.00417.x.

[ece371409-bib-0004] Béziers, P. , and A. Roulin . 2016. “Double Brooding and Offspring Desertion in the Barn Owl *Tyto alba* .” Journal of Avian Biology 47: 235–244. 10.1111/jav.00800.

[ece371409-bib-0005] Fajardo, I. 2001. “Monitoring Non‐Natural Mortality in the Barn Owl ( *Tyto alba* ), as an Indicator of Land Use and Social Awareness in Spain.” Biological Conservation 97, no. 2: 143–149. 10.1016/S0006-3207(00)00091-4.

[ece371409-bib-0006] Golawski, A. , Z. Kasprzykowski , and M. Kowalski . 2003. “The Occurrence of the Barn Owl *Tyto Alba* in Sacred Buildings in Central‐Eastern Poland.” Ornis Hungarica 12–13: 275–277.

[ece371409-bib-0007] Kalotás, Z. 1987. “A gyöngybagoly (*Tyto alba*) 1985 évi országos állományfelmérésének erdményei.” Madártani tájékoztató 1‐2: 7–11.

[ece371409-bib-0008] Klein, Á. , C. László , and R. Mátics . 2022. “Gyöngybagoly (*Tyto alba*).” In Magyarország ragadozó madarai és bagjai 2. kötet. Sólyomalakúak és bagolyalakúak, edited by L. Haraszthy and J. Bagyura , 9–39. Magyar Madártani és Természetvédelmi Egyesület.

[ece371409-bib-0009] Klein, Á. , R. Mátics , and Z. Schneider . 2023. “Breeding and Conservation Status of the Western Barn Owl ( *Tyto alba* ) in Zala County, Hungary. An Overview of 39 Years of Data.” Ornis Hungarica 31, no. 2: 203–216. 10.2478/orhu-2023-0030.

[ece371409-bib-0010] Klein, Á. , T. Nagy , T. Csörgő , and R. Mátics . 2007. “Exterior Nest‐Boxes May Negatively Affect Barn Owl *Tyto Alba* Survival: An Ecological Trap.” Bird Conservation International 17, no. 3: 273–281. 10.1017/S0959270907000792.

[ece371409-bib-0011] Martinez, J. A. , and I. Zuberogoitia . 2004. “Habitat Preferences and Causes of Population Decline for Barn Owls *Tyto Alba*: A Multi‐Scale Approach.” Ardeola 51, no. 2: 303–317.

[ece371409-bib-0012] Mátics, R. 1999. “A gyöngybagoly (*Tyto alba* Scop., 1769) Mortalitása Magyarországon a gyűrűzési adatok tükrében.” Aquila 105‐106: 125–133.

[ece371409-bib-0013] Mátics, R. 2004. “A gyöngybagoly (*Tyto alba*) természetes és nem természetes mortalitása: nő az utakon történő pusztulás jelentősége.” Természetvédelmi Közlemények 11: 517–524.

[ece371409-bib-0014] Newton, I. , I. Wyllie , and L. Dale . 1997. “Mortality Causes in British Barn Owls ( *Tyto alba* ), Based on 1,101 Carcasses Examined During 1963–1996.” In Biology and Conservation of Owls of the Northern Hemisphere: 2nd International Symposium, edited by J. R. Duncan , D. H. Johnson , and T. H. Nicholls Gen. Tech. Rep. NC‐190. St. Paul, MN: U.S. Dept. of Agriculture, Forest Service, North Central Forest Experiment Station. 299–307. https://research.fs.usda.gov/treesearch/15505.

[ece371409-bib-0015] Poprach, K. 2010. The Barn Owl (S. Sweeney, Trans.). TYTO, Nenakonice, Czech Republic.

[ece371409-bib-0016] Ramsden, D. J. 1998. “Effect of Barn Conversions on Local Populations of Barn Owl *Tyto Alba* .” Bird Study 45, no. 1: 68–76. 10.1080/00063659809461079.

[ece371409-bib-0017] Rydell, J. , J. Eklöf , and S. Sánchez‐Navarro . 2017. “Age of Enlightenment: Long‐Term Effects of Outdoor Aesthetic Lights on Bats in Churches.” Royal Society Open Science 4: 4161077. 10.1098/rsos.161077 PMC5579077 28878962

[ece371409-bib-0018] Šálek, M. , K. Poprach , L. Opluštil , D. Melichar , J. Mráz , and R. Václav . 2019. “Assessment of Relative Mortality Rates for Two Rapidly Declining Farmland Owls in The Czech Republic (Central Europe).” European Journal of Wildlife Research 65: 19. 10.1007/s10344-019-1253-y.

[ece371409-bib-0019] Séchaud, R. , K. Schalcher , B. Almasi , et al. 2022. “Home Range Size and Habitat Quality Affect Breeding Success but Not Parental Investment in Barn Owl Males.” Scientific Reports 12: 6516. 10.1038/s41598-022-10324-7. 35444196 PMC9021228

[ece371409-bib-0020] Skórka, P. , M. Żmihorski , E. Grzędzicka , R. Martyka , and W. J. Sutherland . 2018. “The Role of Churches in Maintaining Bird Diversity: A Case Study From Southern Poland.” Biological Conservation 226: 280–287.

[ece371409-bib-0021] Soulsbury, C. D. , and P. C. White . 2015. “Human–Wildlife Interactions in Urban Areas: A Review of Conflicts, Benefits and Opportunities.” Wildlife Research 42, no. 7: 541–553.

[ece371409-bib-0022] Stone, E. , M. R. K. Zeale , S. E. Newson , W. J. Browne , S. Harris , and G. Jones . 2015. “Managing Conflict Between Bats and Humans: The Response of Soprano Pipistrelles ( *Pipistrellus pygmaeus* ) to Exclusion From Roosts in Houses.” PLoS One 10: e0131825. 26244667 10.1371/journal.pone.0131825 PMC4526527

[ece371409-bib-0023] Taylor, I. 2004. Barn Owls: Predator–Prey Relationships and Conservation. Cambridge University Press.

[ece371409-bib-0024] Torres, A. , J. A. G. Jaeger , and J. C. Alonso . 2016. “Assessing Large‐Scale Wildlife Responses to Human Infrastructure Development.” Proceedings of the National Academy of Sciences 113, no. 30: 8472–8477. 10.1073/pnas.1522488113 PMC4968732 27402749

[ece371409-bib-0025] Zeale, M. R. K. , E. Bennitt , S. E. Newson , et al. 2016. “Mitigating the Impact of Bats in Historic Churches: The Response of Natterer's Bats *Myotis Nattereri* to Artificial Roosts and Deterrence.” PLoS One 211, no. 3: e0152531. 10.1371/journal.pone.0146782 PMC4714818 26771548

[ece371409-bib-0026] Żmihorski, M. , M. Kowalski , J. Cichocki , et al. 2020. “The Use of Socio‐Economy in Species Distribution Modelling: Features of Rural Societies Improve Predictions of Barn Owl Occurrence.” Science of the Total Environment 741: 140407. 10.1016/j.scitotenv.2020.140407. 32603947

